# Individual and seasonal determinants of death among influenza patients in intensive care units: a retrospective cohort study, Portugal, 2012 to 2024

**DOI:** 10.2807/1560-7917.ES.2026.31.8.2500505

**Published:** 2026-02-26

**Authors:** Sebastian von Schreeb, Ana Firme, Mariana Ferreira, Carina Castro Silva, Joana Vidal-Castro, Eunice das Neves Salgado Crisóstomo, Catarina Filipa Sousa Marques, Hugo Filipe Baptista Monteiro, Filipe Froes, Rui Pedro Leitão, Kostas Danis, Isabel Marinho Falcão, Paula Vasconcelos, Vasco Ricoca Peixoto

**Affiliations:** 1 Public Health Emergencies Operations Centre, Directorate-General of Health, Lisbon, Portugal; 2 ECDC Fellowship Programme, Field Epidemiology path (EPIET), European Centre for Disease Prevention and Control (ECDC), Stockholm, Sweden; 3 Pulmonology and Intensive Care - Chest Department, Hospital Pulido Valente, Lisbon, Portugal; 4 NOVA National School of Public Health, Public Health Research Centre, Comprehensive Health Research Center, CHRC, NOVA University Lisbon, Lisbon, Portugal

**Keywords:** Intensive Care Units, Severe acute respiratory infections, SARI, Influenza, Human, Health Services Needs and Demand, Mortality

## Abstract

**BACKGROUND:**

Portugal is establishing a surveillance system for severe acute respiratory infection (SARI). Data from its existing influenza surveillance system in intensive care units (ICU), operating since 2012, has not yet been analysed.

**AIM:**

We aimed to identify individual and seasonal determinants of death from influenza in ICUs to inform ICU capacity planning, triage and SARI surveillance.

**METHODS:**

We conducted a retrospective cohort study of laboratory-confirmed influenza cases admitted to 27 ICUs between 2012 and 2024. Covariates included demographics, comorbidities, season, influenza type, seasonal and weekly influenza caseload in the ICU and weekly ICU occupancy. We calculated case fatality rates and adjusted risk ratios (aRR) for death during ICU admission using log-binomial regression. Directed acyclic graphs informed model-specific adjustments, including age, sex, influenza type and comorbidities.

**RESULTS:**

Of 1,071 cases with known outcome, 262 (24%) died. Case fatality rates were higher among patients with chronic liver disease (aRR: 2.0; 95% CI: 1.5–2.6), cancer (aRR: 1.6; 95% CI: 1.1–2.1) and during a high caseload season (aRR: 1.52; 95% CI: 1.16–2.05). Case fatality rates increased with age and were highest for those aged ≥80 years (aRR: 11; 95% CI: 2.4–184), compared with 0–19-year-olds.

**CONCLUSION:**

Liver disease, cancer and older age were associated with increased fatality. Case fatality was higher in seasons with higher caseloads but showed no significant variation within seasons and did not increase during influenza peaks. These findings inform ICU triage and capacity planning for future seasons and support the implementation of broader SARI surveillance.

**Key public health message**

**What did you want to address in this study and why?**

We analysed 12 seasons of Portugal’s influenza intensive care unit (ICU) surveillance data (2012–2024) to learn which patients are most at risk of dying, so hospitals can better decide who needs an ICU bed and to inform ICU capacity planning for future seasons.

**What have we learnt from this study?**

One in four of the 1,071 influenza patients with known outcome died in the ICU. Deaths were more common among patients with higher age, cancer, chronic liver disease and during seasons when the number of influenza cases was higher than usual. Nevertheless, fatality rates did not vary significantly during influenza seasons, even during peak occupation periods, suggesting that ICUs were able to maintain sufficient capacity and consistent admission practices.

**What are the implications of your findings for public health?**

The identified risk factors should be taken into account when prioritising influenza patients for ICU admission and when planning ICU capacity. The establishment of broader severe acute respiratory infections (SARI) surveillance in Portugal could help generate more robust disease severity estimates for both influenza and other circulating respiratory pathogens.

## Introduction

Influenza is a major contributor to global morbidity and mortality, particularly among vulnerable populations including young children, elderly people, individuals with chronic conditions and pregnant people [1]. In the hospital setting, severe influenza is captured by the broader clinical category of severe acute respiratory infection (SARI), defined by the World Health Organization (WHO) as a hospital admission for acute respiratory illness with fever and cough with onset in the previous 10 days [2]. A portion of these patients require intensive care, and the need for intensive care unit (ICU) beds fluctuates markedly between and within seasons as influenza transmissibility and severity change [3].

The COVID-19 pandemic showed that pathogen-specific surveillance cannot capture the full impact on healthcare systems or respond to preparedness and surveillance objectives such as generating timely disease severity estimates. In response, the European Union (EU) legal framework now recognises SARI surveillance of all hospitalised patients as critical to public health emergency preparedness [4]. Portugal is in the process of establishing such a SARI surveillance system, in line with recommendations from the European Centre for Disease Prevention and Control (ECDC) and the WHO [5,6].

While influenza surveillance is well studied at the primary-care level, evidence on ICU surveillance is limited [7], with studies on case fatality showing large heterogeneity [5]. In Portugal, a surveillance system for monitoring influenza in ICUs was set up in 2011–2012 following the 2009 A(H1N1) pandemic. This surveillance is a collaboration between a network of ICUs, the Directorate-General of Health (DGS) and the National Institute of Health (INSA). These surveillance data provide an opportunity to estimate ICU case fatality rates from severe influenza and identify underlying risk factors for death [6]. Furthermore, the data can be used to evaluate the relationship between ICU occupancy and case fatality rates over 12 years, both between and throughout seasons, and to assess whether ICU surveillance data can detect seasonal variations in influenza severity.

To date, no prior study has analysed case-based data from this ICU influenza surveillance system. This study aims to identify individual and seasonal determinants of death among influenza ICU patients between 2012 and 2024, hypothesising higher case fatality rates among older patients, those with comorbidities and during periods of increased ICU caseload. It seeks to provide evidence to inform ICU triage and capacity planning for future seasons and to support the implementation of broader SARI surveillance in Portugal.

## Methodology

### Study design and study population

A retrospective cohort study was conducted to identify determinants of death during ICU admission for influenza. The study population consisted of all laboratory-confirmed influenza cases admitted to an ICU in the network of sentinel ICUs participating in the Portuguese influenza surveillance system. The network includes 27 ICUs from 21 hospitals, covering ca 300 beds, which represents roughly one third of national ICU capacity and covers the five mainland regions and the two autonomous regions [8,9]. Individual level data were reported to the surveillance system by a contact person at each ICU on a weekly basis. We followed up at the end of each influenza season to supplement missing information.

### Testing criteria and case definition

In the ICU surveillance system, patients are tested for influenza based on clinical criteria for acute respiratory infection or influenza-like illness. Acute respiratory infection is defined as sudden onset of symptoms within 24 hours, at least one respiratory symptom (cough, sore throat, rhinorrhoea or dyspnoea) and clinical judgment of infection. Influenza-like illness is defined as sudden onset of symptoms within 24 hours, at least one systemic symptom (fever, malaise, headache or myalgia) and at least one respiratory symptom (cough, sore throat or dyspnoea). An influenza case is defined as a patient with laboratory confirmation of influenza virus infection by reverse transcription (RT)-PCR or viral culture.

### Definition of outcome, patient variables and seasonal variables

The main outcome was death during ICU stay; deaths occurring after ICU discharge were not included. Patient variables included influenza virus type and subtype, age, sex and comorbidities, and were reported by the ICU through standardised case report forms as part of the influenza ICU surveillance system. We defined region according to the geographic location of the reporting ICU. We defined seasons as epidemiological week 26 to week 25 of the following year to reflect the current practice of year-round influenza surveillance. We dichotomised seasonal influenza caseload at the overall median number of influenza ICU admissions per season, defining seasons above the median as high caseload seasons. We defined influenza peak weeks as the 3 consecutive weeks with the highest 3-week moving average of influenza cases in that season. We defined ICU occupancy level as the percentage of maximum seasonal ICU all-cause occupancy in the reporting unit at the time of admission, where 100% corresponds to the week with the highest all cause occupancy in that season.

### Statistical analysis

**Figure 1:**
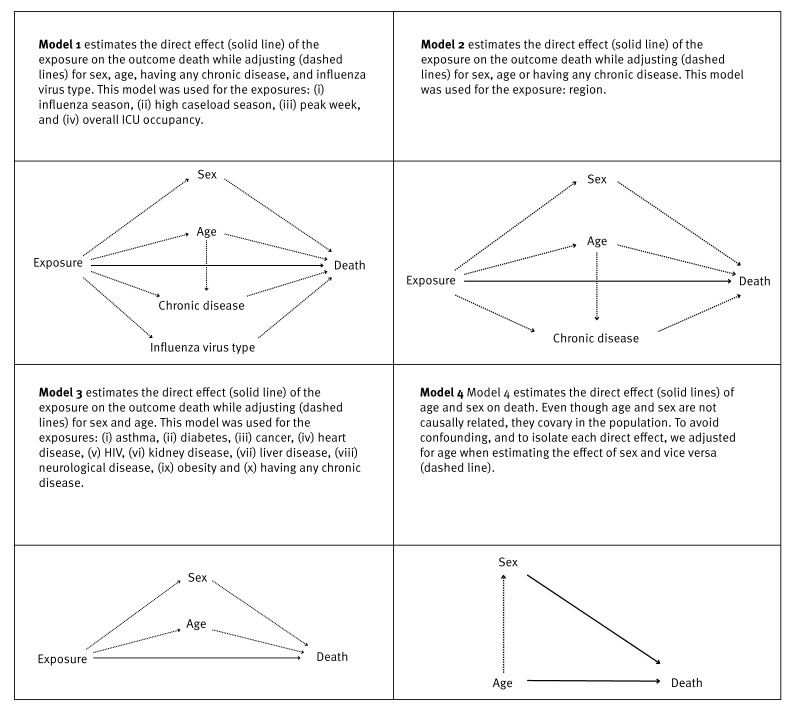
Directed acyclic graph (DAG) illustrating the relationship between exposure (patient variables and seasonal variables) and outcome (death), Portugal, 2012–2024

We calculated case fatality rates and estimated risk ratios (RR) and adjusted risk ratios (aRR) for death using univariable and multivariable log-binomial regression. To account for potential confounding, we used directed acyclic graphs (DAGs) to outline the hypothesised causal relationships for each variable. We then performed multivariable regression, adjusting the exposure (patient variables and seasonal variables) for the confounders specified in the corresponding DAG (Figure 1). Although age and sex are not causally related, they covary in the population and we therefore adjusted for both when estimating their direct effects on death.

ICU: intensive care unit.

The arrows in the DAG illustrate the hypothesised directionality of the relationship between variables.

We performed a sensitivity analysis to evaluate the effect of high caseload seasons by excluding the first two seasons of the COVID-19 pandemic to ensure that the estimate was not caused by the very low caseload during these seasons.

## Results

**Figure 2:**
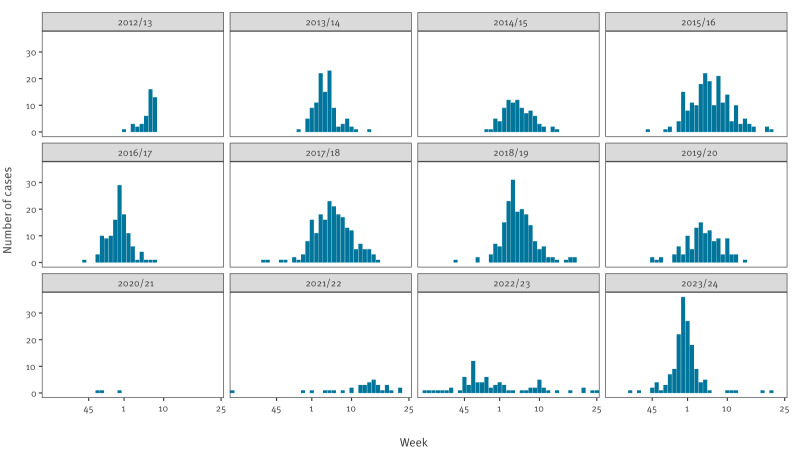
Influenza cases reported to the national sentinel surveillance for influenza in intensive care units, Portugal, 2012–2024 (n = 1,350)

Between 2012 and 2024, 1,350 laboratory-confirmed influenza cases were admitted to the 27 sentinel ICUs (Figure 2). Among the 1,071 (79%) cases with known outcome, 262 died in the ICU, resulting in an overall case fatality rate of 24%.

### Patient characteristics

Compared with cases aged 0–19 years and adjusting for sex, case fatality was 5.5 (95% confidence interval (CI): 1.1–98.0) times higher in those aged 20–39 years, 7.6 (95% CI: 1.8–132.0) times higher in those aged 40–59 years, 9.9 (95% CI: 2.3–172.0) times higher in those aged 60–79 years and 11 (95% CI: 2.4–184.0) times higher among cases aged 80 years or older. Sex was not associated with death (aRR 1.08; 95% CI: 0.88–1.40) (Table 1).

**Table 1:** Descriptive, univariable and multivariable analysis of patient characteristics and risk ratio of fatal outcome among influenza patients admitted to a participating intensive care unit, Portugal, 2012–2024

Characteristics	Descriptives			Univariable			Multivariable		
	Total (n)	Dead (n)	Dead (%)	RR	95% CI	p value	aRR	95% CI	p value
Total	1,071	262	25	NA	NA	NA	NA	NA	NA
Sex^a^									
Female	452	106	24	Reference					
Male	619	156	25	1.07	0.87–1.34	0.500	1.08	0.87–1.34	0.500
Age group (years)^a^									
0–19	129	14	12	Reference					
40–59	335	71	21	1.80	1.10–3.22	***0.031***	1.79	1.09–3.21	***0.032***
60–79	473	133	28	2.39	1.49–4.20	* * ***< 0.001***	2.38	1.49–4.19	* * ***< 0.001***
≥80	142	43	30	2.57	1.53–4.66	* * ***< 0.001***	2.58	1.53–4.68	* * ***< 0.001***
Any chronic disease^a^	857	220	26	1.39	1.04–1.92	***0.035***	1.12	0.83–1.57	0.500
Asthma^a^	44	10	23	0.92	0.49–1.52	0.800	0.96	0.51–1.57	0.900
COPD^a^	163	41	25	1.03	0.75–1.39	0.800	0.96	0.69–1.29	0.800
Diabetes^a^	260	72	28	1.20	0.94–1.50	0.130	1.09	0.85, 1.37	0.500
Cancer^a^	91	34	37	1.66	1.20–2.22	***0.001***	1.57	1.13–2.10	***0.004***
Heart disease^a^	401	107	27	1.17	0.94–1.45	0.200	0.98	0.78–1.23	0.900
HIV infection^a^	12	3	25	1.02	0.28–2.19	> 0.900	1.00	0.27–2.19	> 0.900
Kidney disease^a^	154	49	32	1.39	1.05–1.78	***0.014***	1.24	0.94–1.60	0.110
Liver disease^a^	58	28	48	2.10	1.53–2.74	* * ***< 0.001***	2.01	1.46–2.62	* * ***< 0.001***
Neurological disease^a^	27	10	37	1.55	0.85–2.37	0.090	1.48	0.79–2.30	0.150
Obesity^a,b^	274	56	20	0.79	0.60–1.02	0.082	0.79	0.60–1.02	0.077
Region^c^									
Alentejo	56	8	14	Reference					
Algarve	2	0	0	NA	NA	NA	NA	NA	NA
Azores	55	9	16	1.15	0.47–2.85	0.800	1.17	0.48–2.90	0.700
Central	258	66	26	1.79	0.98–3.86	0.090	1.78	0.98–3.84	0.088
LVT	598	146	24	1.71	0.96–3.63	0.110	1.74	0.98–3.69	0.095
Madeira	37	12	32.4	2.27	1.04–5.30	***0.043***	2.26	1.04–5.25	***0.043***
North	65	21	32.3	2.26	1.14–5.07	***0.029***	2.43	1.23–5.42	***0.016***

aRR: adjusted risk ratio; CI: confidence interval; COPD: chronic obstructive pulmonary disease; LVT: Lisboa e Vale do Tejo; NA: not applicable; Ref: reference category; RR: risk ratio.

^a ^Multivariable model adjusted for age and sex.

^b ^Body mass index over 30.

^c ^Multivariable model adjusted for age, sex and at least one chronic disease.

Bold italics indicate significant difference (p < 0.05).

Chronic liver disease (aRR: 2.00; 95% CI: 1.46–2.60) and cancer (aRR: 1.57; 95% CI: 1.13–2.10) were associated with increased case fatality after adjusting for age and sex. Cases with obesity (body mass index above 30) had a non-significant 22% lower risk of death (aRR: 0.78, 95% CI: 0.59–1.01) compared with other cases. Case fatality was not significantly higher among patients with kidney disease after adjusting for age and sex (aRR: 1.2; 95% CI: 0.94–1.60). Asthma, chronic obstructive pulmonary disease, diabetes, heart disease, HIV infection, neurological disease and obesity were not associated with fatal outcome (Table 1).

Compared with Alentejo, higher case fatality was observed in Madeira (aRR: 2.28; 95% CI: 1.06–5.31) and in the North region (aRR: 2.40; 95% CI: 1.21–5.36). Estimates for the remaining regions (Central, Lisboa e Vale do Tejo, Azores and Algarve) did not differ significantly from Alentejo.

There were no significant differences in case fatality when comparing cases admitted to the ICU with influenza type B to those admitted with influenza type A or influenza type A subtypes (H1 and H3).

### Seasonal characteristics

Compared with the latest season at the time of writing (2023/24), case fatality was markedly lower in 2022/23 (aRR: 0.46; 95% CI: 0.23–0.91). No significant differences were detected for seasons from 2012/13 to 2019/20 compared with 2023/24 (Table 2). Risk ratios were not calculated for seasons 2020/21 and 2021/22 as no deaths were reported during those seasons which coincided with the COVID-19 pandemic.

**Table 2:** Descriptive, univariable and multivariable analysis of seasonal variables and risk ratios of fatal outcome among influenza patients admitted to a participating intensive care unit, Portugal, 2012–2024

Characteristic	Descriptives			Univariable			Multivariable		
	Total (n)	Dead (n)	Dead (%)	RR	95% CI	p value	RR	95% CI	p value
Total	1,071	262	24.5	NA	NA	NA	NA	NA	NA
Influenza peak weeks^a^									
No	624	168	26.9	Reference					
Yes	404	90	22.3	0.83	0.66–1.03	0.100	0.82	0.66–1.03	0.088
ICU occupancy level, median (Q1–Q3)^a^	88 (81–92)	88 (81–92)	NA	1.00	0.98–1.01	> 0.900	1.00	0.98–1.01	0.800
High caseload season^a^									
No	283	49	17	Reference					
Yes	788	213	27	1.56	1.19–2.09	***0.002***	1.52	1.16–2.05	***0.004***
Season^b^									
2023/24	144	40	27.8	Reference					
2022/23	64	8	12.5	0.45	0.21–0.85	***0.025***	0.46	0.23–0.91	***0.026***
2021/22	30	0	0	NA	NA	NA	NA	NA	NA
2020/21	2	0	0	NA	NA	NA	NA	NA	NA
2019/20	90	21	23.3	0.84	0.52–1.31	0.500	0.87	0.55–1.37	0.500
2018/19	164	49	29.9	1.08	0.76–1.54	0.700	1.08	0.76–1.53	0.700
2017/18	180	47	26.1	0.94	0.66–1.35	0.700	0.80	0.53–1.21	0.300
2016/17	84	23	27.4	0.99	0.62–1.51	> 0.900	0.79	0.50–1.27	0.300
2015/16	126	33	26.2	0.94	0.63–1.40	0.800	1.07	0.72–1.59	0.800
2014/15	91	21	23.1	0.83	0.51–1.30	0.400	0.76	0.47–1.24	0.300
2013/14	85	16	18.8	0.68	0.39–1.11	0.140	0.72	0.42–1.23	0.200
2012/13	11	4	36.4	1.31	0.46–2.55	0.500	1.38	0.55–3.51	0.500
Influenza type or subtype									
B	158	45	28.5	Reference					
A non-subtyped	403	91	22.6	0.79	0.59–1.09	0.140	0.78	0.58–1.06	0.110
A(H1)	363	94	25.9	0.91	0.68–1.24	0.500	1.03	0.76–1.40	0.800
A(H3)	136	28	20.6	0.72	0.47–1.08	0.120	0.70	0.46–1.05	0.083
Unspecified	11	4	36.4	1.28	0.45–2.46	0.600	1.40	0.65–3.00	0.400

CI: confidence interval; ICU: intensive care unit; NA: not applicable; Q: quartile; RR: risk ratio.

^a ^Multivariable model adjusted for age, sex and any chronic disease.

^b ^Multivariable model adjusted for age, sex, any chronic disease and virus type.

‘Influenza peak weeks’ refers to admission during the seasonal peak, defined as the 3 consecutive weeks with the highest 3-week moving average of cases.

‘ICU occupancy level’ refers to the percentage of maximum seasonal ICU occupancy (for all causes) in the reporting unit at the time of admission, where 100% corresponds to the week with the highest occupancy in that season.

‘High caseload season’ refers to seasons with total influenza ICU admissions above the seasonal median.

Bold italics indicate significant difference (p < 0.05).

Case fatality in high caseload seasons was 38% higher compared with nonhigh caseload seasons (aRR: 1.38; 95% CI: 1.10–1.70). When we excluded the two seasons with very low caseloads during the COVID-19 pandemic (2020/21 and 2021/22), case fatality remained higher in high caseload seasons (aRR: 1.28, 95% CI: 1.01–1.70).

Throughout the seasons, case fatality did not significantly change during the weekly influenza peak (aRR: 0.82; 95% CI: 0.66–1.03). Weekly ICU occupancy at admission was not associated with the outcome (aRR: 1.00; 95% CI: 0.98–1.01).

## Discussion

In this analysis of influenza patients in Portugal’s national surveillance system for influenza in ICUs, the case fatality rate was 24%, consistent with the pooled mortality estimate (24%) from a systematic review of comparable studies across Europe [10].

Case fatality rate increased with increasing age and was higher among patients with chronic liver disease and cancer, after adjusting for age and sex. A similar European study with comparable overall ICU mortality (21%) also found increased risk for these conditions, with comparable risk estimates [6]. A 2019 study from Spain reported that only 18–19% of influenza patients aged 65 years or older were admitted to an ICU compared with 46% of 18–64-year-olds [11]. If triage practices are similar in Portugal, the increased case fatality rate we found for older patients likely underestimates the true difference between age groups, as severely ill older adults may be less likely than younger patients to be admitted to the ICU due to personal choice [11], frailty or limited expected benefit from intensive care.

Considering comorbidities, the study from Spain reported the highest ICU admission rates among influenza patients with liver disease (46% admitted) and obesity (44%), while the overall admission rate was 34%, suggesting that patients with these conditions were prioritised in ICU triage [11]. To optimise ICU triage of influenza patients, future studies should compare the case fatality rates observed in this study with comorbidity-specific ICU admission rates in Portugal, particularly for liver disease, kidney disease, cancer and obesity. This comparison may help evaluate whether these patient groups are under- or over-admitted in relation to their risk of death, thereby optimising the use of ICU resources [5].

In the 2020/21, 2021/22 and 2022/23 seasons, influenza ICU admissions and case fatality rates were lower than in other seasons, likely due to shifts in healthcare prioritisation and reduced influenza circulation during the COVID-19 pandemic [12,13]. In 2023/24, case fatality rates returned to pre-pandemic levels, suggesting a normalisation of outcomes among ICU influenza patients in Portugal.

High caseload seasons were associated with higher fatality rates after adjusting for sex, age, having a chronic disease and influenza virus type. This association remained statistically significant in the sensitivity analysis when we excluded the two very low caseload seasons during the COVID-19 pandemic, although the lower confidence bound was closer to one, indicating higher uncertainty about the result. To better understand if high ICU occupancy is associated with higher case fatality, as proposed by previous studies [14], we estimated the effect of being admitted during influenza peak weeks, or in weeks with high overall ICU burden. However, none of these variables were associated with increased fatality. A possible explanation is the smaller sample size in our study. A 2010 study in the United States (US) with 166,920 hospitalised patients found a significant association between overall case fatality and admission during a period with widespread influenza, as defined by the US Centers for Disease Control and Prevention (CDC). However, the absolute difference in case fatality was only 0.5 percentage points higher compared with periods without widespread influenza [15], which may have been too small for our study to detect. Taken together, our results suggest consistent ICU triage and management practices throughout the seasons analysed in Portugal. Future studies should investigate other potential underlying factors that could explain the association between high caseload seasons and case fatality.

We also observed differences in case fatality rates between regions, ranging from 14 to 32%. These differences should be interpreted with caution as they may be influenced by factors such as admission practices affecting the numerator, and patient transfer patterns between hospitals according to ICU capacity, which alters the distribution of severe cases between ICUs. No significant difference in mortality was observed between influenza types or subtypes, although point estimates for A(H3) and non-subtyped influenza A suggested lower case fatality compared with influenza B. The wide and overlapping confidence intervals indicate limited precision and no clear evidence of variation in outcome by type/subtype.

Overall, the presented findings reinforce the importance of expanding surveillance efforts beyond ICUs to better capture severity differences between seasons and patient groups. As recommended by ECDC and the WHO [10,16], Portugal is in the process of extending the current surveillance system to include all hospital-admitted SARI patients using the Portuguese Hospital Morbidity Database and International Classification of Diseases (ICD-10) codes for diagnoses, procedures and outcomes. Future analysis using the broader SARI surveillance will allow for better pathogen-specific severity estimates to detect early signs of increased severity of the monitored pathogen. These estimates are important for scenario modelling to inform preventive measures to reduce transmission and improve hospital capacity planning. The Commission Implementing Regulation (EU) 2023/1808 emphasises the critical role of SARI surveillance in informing prevention, preparedness and response planning for serious cross-border threats to health in accordance with Regulation (EU) 2022/2371 of the European Parliament and of the Council [17].

The present study had several limitations. First, ICU patients were admitted based on clinical severity and other contextual criteria, and thus they do not represent a random sample of patients with severe influenza. As such, the associations observed may reflect both underlying risks and selection practices for ICU admission. However, the overall case fatality rate was closely aligned with pooled estimates of previous research [5] and ICU data remain valuable for identifying predictors of increased risk. The use of SARI surveillance data of all hospitalised patients will reduce the risk of selection bias in disease severity estimates. Second, participating ICUs may not be representative of all ICUs in Portugal due to voluntary unit recruitment and accuracy of ICU occupancy data may vary across reporting units. Third, the surveillance system does not distinguish whether influenza was the primary reason for ICU admission and testing is influenced by clinical suspicion. However, the surveillance reporting is designed for patients with symptomatic influenza and not for asymptomatic influenza detected through screening, and there were no normative changes in testing practices throughout the study period. Nevertheless, this may introduce bias, for example if younger patients with clearer symptoms are more likely to be tested than older patients with atypical presentations. Fourth, comorbidity data were reported as binary variables without details on severity or treatment status, which may have introduced confounding. Fifth, vaccination status was not included in the final models due to incomplete reporting, which limits the assessment of its protective effect. We were also unable adjust for vaccination as a potential confounder for the variables examined in this study, particularly among groups targeted by the national vaccination recommendations, including individuals with comorbidities and people aged over 65 years, and, since the 2023/24 season, those aged 60–64 years. Vaccination data linkage should be included in the developing SARI surveillance system. Sixth, therapeutic interventions, ventilation, extracorporeal membrane oxygenation, antiviral use, renal replacement therapy and others are possibly unobserved confounders. Finally, the outcome was limited to deaths during ICU stay and did not capture deaths occurring after discharge.

## Conclusions

The current study identified both individual and seasonal factors associated with risk of death among influenza patients admitted to the ICU. The risk of death was higher among patients with cancer, chronic liver disease or older age, which should be accounted for in ICU triage. High caseload seasons had higher case fatality rates. However, fatality rates did not significantly differ during seasonal peak weeks or at times of high ICU occupancy, suggesting consistent triage practices. Capacity planning should account for increased fatality rates during high caseload seasons, and underlying factors beyond ICU occupancy should be explored. The establishment of a national SARI surveillance system is underway and should build on this evidence by incorporating pathogen-specific severity indicators, enabling earlier detection of increased severity and comparison of case fatality rates with ICU admission rates to better understand triage practices.

## Data Availability

The R code used for analysis is available upon reasonable request to the corresponding author.
